# Electron Beam-Treated Enzymatically Mineralized Gelatin Hydrogels for Bone Tissue Engineering

**DOI:** 10.3390/jfb12040057

**Published:** 2021-10-08

**Authors:** Stefanie Riedel, Daniel Ward, Radmila Kudláčková, Karolina Mazur, Lucie Bačáková, Jemma G. Kerns, Sarah L. Allinson, Lorna Ashton, Robert Koniezcny, Stefan G. Mayr, Timothy E. L. Douglas

**Affiliations:** 1 Leibniz Institute of Surface Engineering (IOM), Permoserstraße 15, 04318 Leipzig, Germany; robert.konieczny@iom-leipzig.de (R.K.); stefan.mayr@iom-leipzig.de (S.G.M.); 2 Division of Surface Physics, Faculty of Physics and Earth Science, Leipzig University, Linnéstraße 5, 04103 Leipzig, Germany; 3 Division of Biomedical and Life Sciences (BLS), Faculty of Health and Medicine, Furness College, Lancaster University, Lancaster LA1 4YG, UK; dannywlmr9@hotmail.co.uk (D.W.); s.allinson@lancaster.ac.uk (S.L.A.); 4 Department of Biomaterials and Tissue Engineering, Institute of Physiology of the Czech Academy of Sciences, Vídeňská 1083, 142 00 Prague, Czech Republic; KudlackovaRadmila@seznam.cz (R.K.); Lucie.Bacakova@fgu.cas.cz (L.B.); 5 Faculty of Materials Engineering and Physics, Institute of Materials Engineering, Tadeusz Kosciuszko Cracow University of Technology, al. Jana Pawła II 37, 31-864 Cracow, Poland; mazur117@o2.pl; 6 Lancaster Medical School, Faculty of Health and Medicine, Lancaster University, Lancaster LA1 4YW, UK; j.kerns@lancaster.ac.uk; 7 Chemistry Department, Lancaster University, Lancaster LA1 4YB, UK; l.ashton@lancaster.ac.uk; 8 Engineering Department, Lancaster University, Lancaster LA1 4YW, UK; 9 Materials Science Institute (MSI), Lancaster University, Lancaster LA1 4YW, UK

**Keywords:** bone tissue engineering, enzymatic mineralisation, gelatin hydrogels, electron beam treatment

## Abstract

Biological hydrogels are highly promising materials for bone tissue engineering (BTE) due to their high biocompatibility and biomimetic characteristics. However, for advanced and customized BTE, precise tools for material stabilization and tuning material properties are desired while optimal mineralisation must be ensured. Therefore, reagent-free crosslinking techniques such as high energy electron beam treatment promise effective material modifications without formation of cytotoxic by-products. In the case of the hydrogel gelatin, electron beam crosslinking further induces thermal stability enabling biomedical application at physiological temperatures. In the case of enzymatic mineralisation, induced by Alkaline Phosphatase (ALP) and mediated by Calcium Glycerophosphate (CaGP), it is necessary to investigate if electron beam treatment before mineralisation has an influence on the enzymatic activity and thus affects the mineralisation process. The presented study investigates electron beam-treated gelatin hydrogels with previously incorporated ALP and successive mineralisation via incubation in a medium containing CaGP. It could be shown that electron beam treatment optimally maintains enzymatic activity of ALP which allows mineralisation. Furthermore, the precise tuning of material properties such as increasing compressive modulus is possible. This study characterizes the mineralised hydrogels in terms of mineral formation and demonstrates the formation of CaP in dependence of ALP concentration and electron dose. Furthermore, investigations of uniaxial compression stability indicate increased compression moduli for mineralised electron beam-treated gelatin hydrogels. In summary, electron beam-treated mineralized gelatin hydrogels reveal good cytocompatibility for MG-63 osteoblast like cells indicating a high potential for BTE applications.

## 1. Introduction

Hydrogels are highly promising materials for tissue engineering applications as they demonstrate biocompatibility, biodegradability and biomimetic properties. However, in the case of bone tissue engineering (BTE), hydrogels have intrinsic disadvantages as they are generally not mineralised with calcium phosphate (CaP), which forms an obstacle to durable interactions with bone tissue. A common strategy to overcome this problem is the incorporation of inorganic particles into the hydrogel matrices such as ceramic CaP, where the particles act as nucleation sites and induce mineralisation [1]. However, this process usually results in the formation of CaP particle aggregates which does not lead to optimal biomimetic properties and local weaknesses in the hydrogel structure and integrity. Therefore, it is necessary to develop techniques and methods to improve the mineralisation process, such as the active formation of CaP within the hydrogel [2], or via utilisation of dispersants such as citrate [3].

Optimizing the mineralization of biological hydrogels is of high importance for BTE, as this process reveals a multitude of advantages [1]. In the presence of CaP mineral, the biological performance of bone-substituting hydrogel materials can be enhanced due to bioactivity, or the formation of direct chemical bonds between the adjacent bone tissue and the implanted material [4]. In addition, CaP ceramics have an intrinsic affinity for bio-active proteins such as growth factors, which are necessary for the natural processes such as healing of bone tissue [5]. Furthermore, as already mentioned, the weak mechanical properties are one of the main disadvantages of hydrogels, which means that their applications without reinforcement are more limited to soft tissue engineering. However, due to the mechanical reinforcement caused by the mineralization, they can be considered for the purposes of hard tissue engineering, such as the regeneration of bone tissue [6]. Mineralisation is further assumed to improve bone tissue-compatibility of hydrogels as rougher and stiffer surfaces are known to promote cell differentiation towards an osteoblastic phenotype. Therefore, the development of precisely adjusted mineralised hydrogels is desirable. The combination of enzymatic mineralisation with precise tailoring methods is of special interest. The presented work aims to combine the advantages of electron beam treatment of gelatin hydrogels, such as chemical-free sterilization, stabilization as well as tuning of material properties, with advantages of enzymatic mineralisation via ALP-mediated by CaGP, such as mechanical reinforcement, increased bioactivity. With a successful ALP-induced mineralisation of electron beam-treated gelatin hydrogels, highly promising materials for BTE can be developed.

## 2. Materials and Methods

### 2.1. Hydrogel Preparation

Gelatin type I (G2500; Sigma-Aldrich Chemie GmbH, Schnelldorf, Germany) was dissolved in ultrapure water (ddH_2_O) with a final concentration of 8 mg/mL. Alkaline phosphatase (ALP; P7640; Sigma-Aldrich, Taufkirchen, Germany) was added to achieve concentrations of 0, 1.25, or 2.5 mg/mL. The gelatin-ALP solution was allowed to swell for one hour at room temperature before being heated to 37 °C. Thereby, simultaneous stirring ensured homogenous dissolution. The solution was left for polymerisation for 12 h at 6 °C.

### 2.2. Electron Beam Treatment

Electron beam irradiation was carried out by a linear electron accelerator (MB10-30MP; Mevex, Stittsville, ON, Canada) with either 40 kGy or doses ranging from 5 to 40 kGy, achieved in steps of 5 kGy. The electron accelerator was operated with a scanning frequency of 3 Hz, and an electron pulse repetition rate of 180 Hz and a pulse length of 8 µm. To avoid radical scavenging due to ambient oxygen, the samples were irradiated in a gaseous nitrogen environment. During the irradiation, samples were cooled via air draft to prevent overheating. The irradiation dose was determined by a graphite dosimeter to an accuracy of 10%.

### 2.3. Enzymatic Mineralization

The hydrogels were mineralised in accordance to Douglas et al. [1]. Therefore, the gelatin hydrogels were incubated in a mineralisation medium containing 0.1 M calcium glycerophosphate (CaGP) in ddH_2_O for 6 days. The mineralisation medium was changed every 24 h. Afterwards, ddH_2_O was used to rinse the samples three times, before incubating them for 24 h to remove residual CaGP.

### 2.4. Scanning Electron Microscopy (SEM)

SEM analysis was performed using a 7800F SEM instrument (JOEL Ltd., Tokyo, Japan). Therefore, samples were positioned on aluminium stubs (Agar Scientific Ltd., London, UK) using carbon adhesive pads (Agar Scientific Ltd., UK). Afterwards, they were coated with a thin layer of gold (~10 nm) using a Q150RES sputter coater (Quorum Technologies Ltd., Laughton, UK).

### 2.5. Raman Spectroscopy

Raman spectra were obtained using a Renishaw InVia Raman Spectrometer (Renishaw Plc., Wotton-under-Edge, UK) utilising an excitation wavelength of 785 nm and 50x objective. The laser power was set to ~15 mW at the sample. Spectral acquisition time was 5 s × 12 repeats. For each sample, three spectra were collected. One sample per experimental condition was investigated. Cosmic ray removal, baseline correction and smoothing were carried out using MATLAB software (The MathWorks, Natick, MA, USA). Smoothing was applied utilising a triangular sliding average. Baseline correction was applied via an asymmetric least squares algorithm.

### 2.6. Inductively Coupled Plasma Optical Emission Spectrometry (ICP-OES)

The calcium (Ca) and phosphorus (P) concentrations as well as the Ca to P ratio, were determined using a 5100 Synchronous Vertical Dual View optical emission spectrometer (Agilent Technologies, Santa Clara, CA, USA). Therefore, hydrogels were dried, dissolved in 3 mL of 14 M HNO_3_ (Fisher Scientific, Loughborough, UK), then further diluted (1:100) using 0.3 M HNO_3_. Standard solutions, with Ca (Fisher Scientific, UK) and P (Alfa Aesar, Heysham, UK) with concentrations ranging from 0 to 250 mg/l were used for calibration. Additionally, all solutions contained Yttrium (Sigma-Aldrich, St. Louis, MO, USA), which was used as an internal standard to account for exogenous effects. Measurements were taken in triplicate. For statistical analysis, one-way ANOVA was performed.

### 2.7. Dry Mass

The ratio of mineralised material was determined via the dry mass percentage. Therefore, samples were weighed in the hydrated ($m_{h}$) and dried state ($m_{d}$). For the latter, the samples were dried for 24 h at 60 °C. Dry mass ratio was then calculated using the following equation, as in previous work [1]:

(1) $$\mathrm{dry}\;\mathrm{mass}=\frac{m_{d}}{m_{h}}\;,$$

To determine the mass percentage of mineral formed in gelatin hydrogels with ALP concentrations of 1.25 and 2.5 mg/mL, at electron irradiation doses ranging from 0 to 40 kGy, mineralised for 6 days in CaGP, the difference between the dry mass of mineralised (1.25 and 2.5 mg/mL ALP in CaGP) and unmineralized (0 mg/mL ALP in CaGP) were calculated. The measurements were performed in triplicate with three independent samples per condition. Mean and standard deviation were determined.

### 2.8. Uniaxial Compression Modulus

The uniaxial compression modulus of the samples in wet state was determined using a universal testing machine (Inspekt mini, Hegewald & Peschke Meß- und Prüftechnik GmbH, Nossen, Germany), with a 100 N load cell. The displacement rate was 2 mm/min until sample failure was reached. Uniaxial compressive stress ($\sigma_{c}$) was determined using the applied force (F) and the cross-sectional area of the sample (A):

(2) $$\sigma_{c}=F/A\;,$$

The uniaxial compression modulus was set as the gradient of the linear-elastic range of the stress–strain curve for small strains. The measurements were performed in triplicate with three independent samples per condition. Mean and standard deviation were determined.

### 2.9. Cytocompatibility

#### 2.9.1. Sample Preparation and Cell Seeding

A human osteosarcoma (MG-63) cell line (Sigma-Aldrich, USA) was cultured on the hydrogels. Hydrogels were cut into slices of 2 mm thickness and placed into 48-well plate (Corning Incorporated, Corning, NY, USA). Subsequently, 5263 cells/cm^2^ were seeded on the materials in Dulbecco’s modified Eagle’s medium (DMEM; Sigma-Aldrich, USA) supplemented with 10% fetal bovine serum (FBS; Thermo Fisher Scientific, Waltham, MA, USA) and Penicillin/Streptomycin (100 IU/mL, 100 μg/mL, respectively). Cells were cultured at 37 °C, with 90% humidity and 5% atmospheric pressure of carbon dioxide (CO_2_).

#### 2.9.2. Fluorescence Microscopy

Cell adhesion and proliferation on materials were evaluated by fluorescence staining on days 1, 4 and 7. Samples were rinsed with phosphate-buffered saline (PBS) and fixed with 70% ethanol (−20 °C) for 10 min. Samples were rinsed with PBS and stained with Hoechst #33,342 (Sigma-Aldrich, USA) with a final concentration of 50 ng/mL and Texas Red C2 maleimide (Invitrogen, Waltham, MA, USA) of final concentration 0.5 µg/mL for 15 min in dark. (Texas Red C2 Maleimide stains proteins of the cell membrane and cytoplasm; maleimide binds to thiol groups, which are present on proteins in cells, either in cytoplasm or in plasma membrane, and is conjugated with a rhodamine derivative, Texas Red, a red fluorescent dye). After staining, samples were rinsed twice with PBS and visualised on an Axio Scope.A1 microscope (Zeiss, Oberkochen, Germany). The images were used for evaluating the cell morphology and distribution, and also the cell number by counting cell nuclei on the images with the following recalculation of these values into the cell population density per cm^2^.

#### 2.9.3. Proliferation Assay (MTS)

In addition to direct counting cells on the materials, the MTS assay was used to determine the proliferation of MG-63 cells on days 1, 4 and 7. The reason was that the cells were often distributed non-homogeneously on the material surface due to the surface irregularities, or were overlapping one another, especially in later culture intervals. DMEM with 10% FBS and Penicillin/Streptomycin was used to dilute the MTS Cell Titer 96 Aqueous One solution cell proliferation assay kit (Promega, Madison, WI, USA) by factor of 1:6. Three independent samples for each sample group and time interval were measured. Tissue culture polystyrene well bottoms served as a control group (PS; Corning Incorporated, USA). To obtain a background of the material, one sample without cells for each sample group and time interval was measured. First, samples were rinsed with PBS, transferred to a new well plate to prevent interference from cells attached to the bottom of a well, and incubated for 2 h at 37 °C in 0.5 mL of the diluted MTS solution. Next, 100 µL of the solution from each well was transferred in triplicate to a 96-well plate to allow the extent of the yellow tetrazolium dye reduction to violet formazan to be quantified by a spectrophotometer (Tecan Infinite M200 Pro, Männedorf, Switzerland), at a wavelength 490 nm. For statistical analysis, one-way ANOVA (SigmaStat 3.5, Systat Software Inc., San Jose, CA, USA) was performed.

## 3. Results

### 3.1. Scanning Electron Microscopy (SEM)

**Figure 1:**
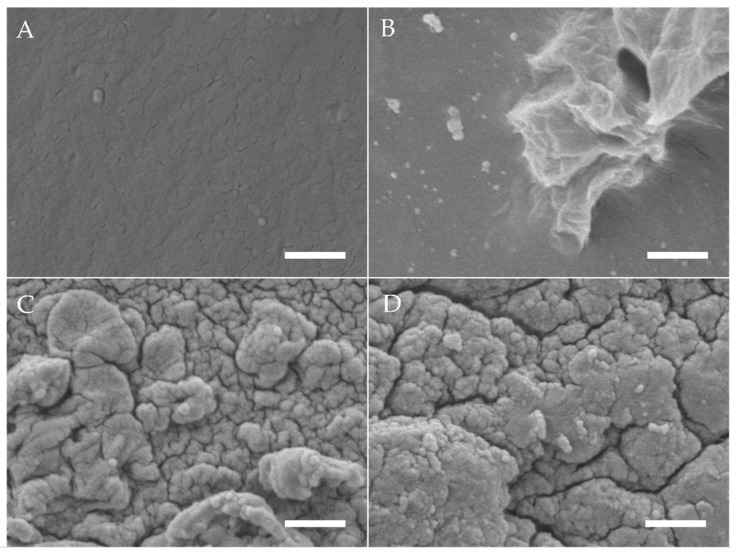
SEM images of gelatin hydrogels (electron beam-treated with 40 kGy) after mineralisation with ALP concentrations of (**A**) 0 mg/mL in ddH_2_O, (**B**) 0, (**C**) 1.25 and (**D**) 2.5 mg/mL incubated for 6 days in CaGP. Scale bars indicate 1 µm.

Using SEM, differences in surface topography of electron beam-treated gelatine hydrogels (40 kGy) with ALP concentrations of 0, 1.25 and 2.5 mg/mL were analysed after 6 days of mineralization in CaGP (Figure 1). The gelatin control samples (unmineralized and ALP/CaGP-free) show a uniformly smooth surface topography. The surfaces of the samples with 0 mg/mL ALP in CaGP are also relatively smooth in appearance. However, their surface display a less uniform morphology as sporadic deposits and inhomogeneous sections can be observed. Despite this, both ALP-free sample groups differ significantly from the ALP-mineralised samples presenting a rougher surface morphology. In general, SEM imaging of both 1.25 and 2.5 mg/mL ALP samples demonstrated successful ALP-induced enzymatic mineralisation. Mineral deposits of irregular shape and size were tightly compact and homogeneously distributed over the surface of the gelatin samples with characteristic diameters of 100 to 500 nm. No obvious correlation between ALP concentration and mineral deposit size was detected.

### 3.2. Raman Spectroscopy

**Figure 2:**
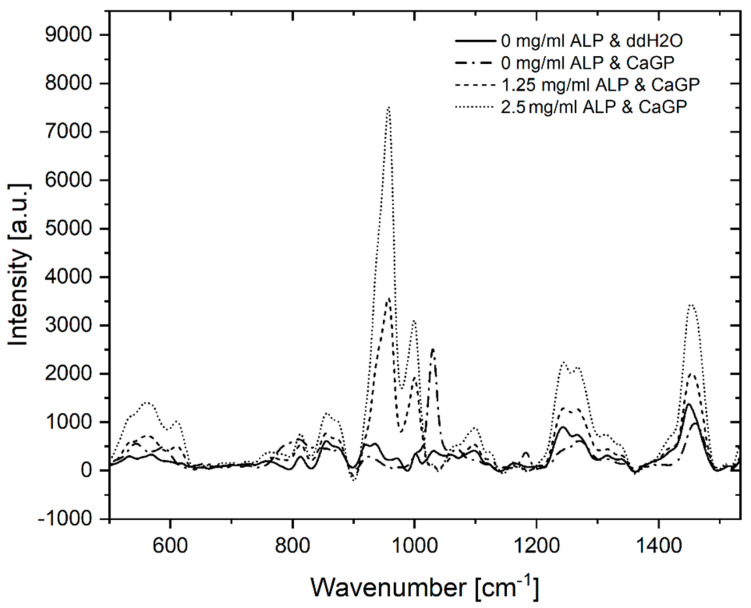
Raman spectra of gelatin hydrogels electron irradiated at doses of 40 kGy, with ALP concentrations of 0, 1.25 and 2.5 mg/mL, subjected to enzymatic mineralisation (in CaGP) for 6 days. Control sample (0 mg/mL ALP) was stored in ddH_2_O.

Raman spectra of gelatin hydrogels with ALP concentrations of 0, 1.25 and 2.5 mg/mL, electron irradiated at 40 kGy, following 6 days of enzymatic mineralization in CaGP, compared to electron beam-treated gelatin hydrogels without ALP stored in ddH_2_O for 6 days, are shown in Figure 2. Modification of hydrogels with ALP changed the appearance of the Raman spectra compared to the spectra of unmodified material. High intensity bands at 957 cm^−1^ and 1000 cm^−1^, which are typical of PO_4_^3−^ groups, were detected for both gelatin samples containing ALP (1.25 and 2.5 mg/mL ALP in CaGP) while the spectra for the 0 mg/mL group were unremarkable, except for a relatively low intensity peak at ~1000 cm^−1^, typical of _v3_PO_4_^3−^ symmetric stretching modes. When the spectra of ALP concentrations from 1.25 to 2.5 mg/mL were compared, the intensity of the _v1_PO_4_^3−^ assigned band at 957 cm^−1^ approximately doubled [7]. Bands at ~560 and ~609 cm^−1^ that correspond to _v4_PO_4_^3−^ bending modes also show an increase in intensity with mineralisation [8]. Additional bands were detected at ~1243 and ~1446 cm^−1^, which are characteristic for Amide III groups [9] (specifically protein β-sheet and less-ordered structures) and protein CH_2_ deformation [10], respectively. Both bands are more intense for 2.5 mg/mL ALP samples compared to 1.25 mg/mL ALP samples. Further bands characteristic of proline [11] and leucine [12] were detected at ~853 cm^−1^.

### 3.3. Inductively Coupled Plasma Optical Emission Spectrometry (ICP-OES)

**Figure 3:**
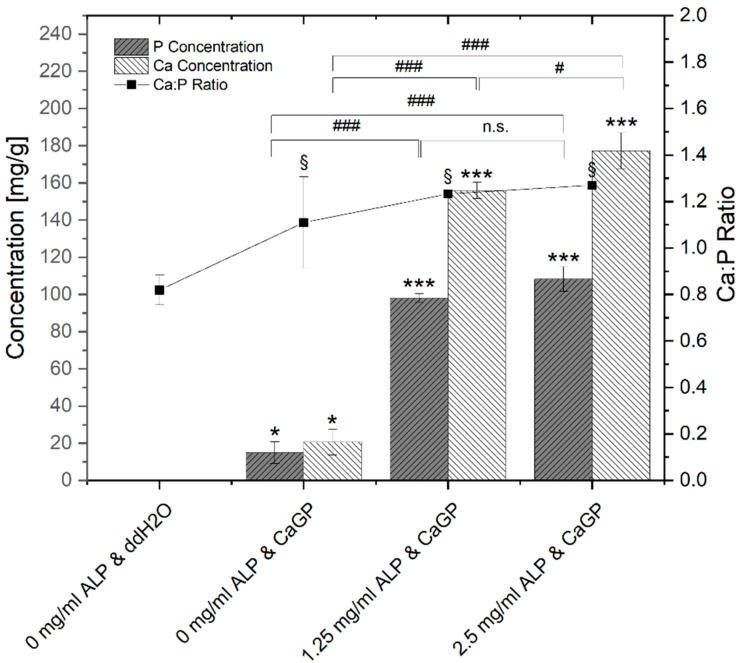
ICP-OES determination of P and Ca concentrations and Ca:P molar ratio of gelatin hydrogels with ALP concentrations of 0, 1.25 and 2.5 mg/mL, electron irradiated with 40 kGy, following 6 days of incubation in ddH_2_O or CaGP. One-way ANOVA was performed. Results expressed as means and standard deviations. Differences considered significant if *p* ≤ 0.05. ‘*’ and ‘§’ relates to the significant difference between groups exposed to CaGP and the ddH_2_O control (* *p* < 0.05, *** *p* < 0.001, § *p* < 0.05). ‘#’ relates to the significant difference between experimental groups (# *p* < 0.05, ### *p* < 0.001).

Results of ICP-OES determination of phosphorus (P) and calcium (Ca) concentrations of gelatin hydrogels with ALP concentrations of 0, 1.25 and 2.5 mg/mL, electron irradiated at 40 kGy, following 6 days of enzymatic mineralization in CaGP, compared to electron beam-treated gelatin hydrogels without ALP stored in ddH_2_O for 6 days, are shown in Figure 3.

Negligible traces of elemental P are detected in the gelatin control group (ALP/CaGP-free). The elemental P concentration was statistically significant increased (*p* < 0.05) when the ddH_2_O was replaced with CaGP, even for the 0 mg/mL ALP samples. With increasing the concentration of ALP to 1.25 and 2.5 mg/mL, statistically significant increases (*p* < 0.001) in the elemental P concentration were observed. Although the observed P concentration of 2.5 mg/mL ALP samples was higher compared to 1.25 mg/mL ALP samples, this increase was not statistically significant.

This trend was also observed for the concentration of elemental Ca. A negligible concentration of Ca was detected in 0 mg/mL ALP in ddH_2_O samples. The concentration increased in a statistically significant manner (*p* < 0.05) in 0 mg/mL ALP in CaGP samples. When ALP concentrations were raised to 1.25 and further to 2.5 mg/mL, mean Ca concentrations also displayed statistically significant increases (*p* < 0.001).

The molar Ca:P ratio determined via ICP-OES is also illustrated in Figure 3. In comparison to stoichiometric hydroxyapatite (Ca:P ratio of 1.67), all detected Ca:P ratios were lower. ALP/CaGP-free samples show a mean Ca:P ratio of 0.82. The ratio increased to 1.11 in 0 mg/mL ALP samples incubated in CaGP and increased further in samples containing 1.25 and 2.5 mg/mL ALP (1.23 and 1.27, respectively), all of which show statistical significance (*p* < 0.05) compared to the control sample (ALP/CaGP-free). However, differences in the molar ratios between the CaGP samples showed no statistical significance.

### 3.4. Dry Mass

**Figure 4:**
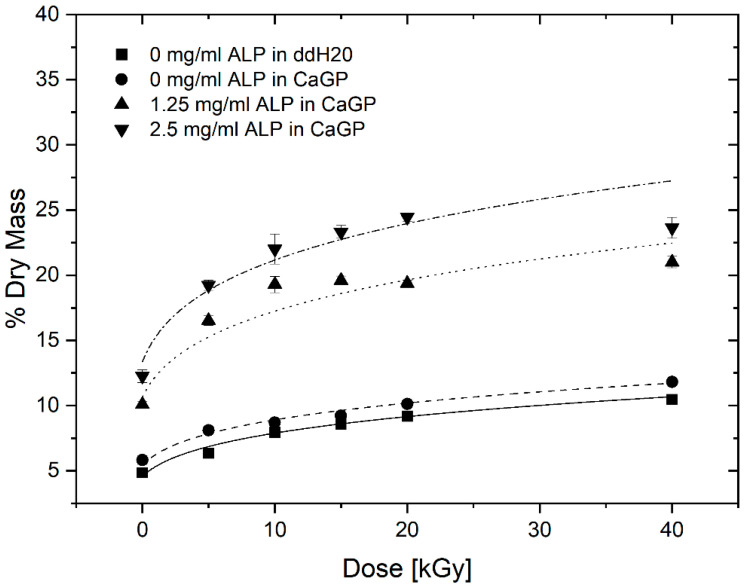
Dry mass of gelatin hydrogels with ALP concentrations of 0, 1.25 and 2.5 mg/mL, at electron irradiation doses ranging from 0 to 40 kGy, mineralised for 6 days in either ddH_2_O or CaGP. Datapoints are fitted with power law fit. Results represent mean values. Error bars show standard deviation.

The percentage of dry mass for gelatin hydrogels with ALP concentrations of 0, 1.25 and 2.5 mg/mL, exposed to radiation doses of 0, 5, 10, 15, 20 and 40 kGy, incubated for 6 days in CaGP and ddH_2_O (control), is illustrated in Figure 4. The dry mass percentage of samples increased with larger concentration of ALP and increasing irradiation dose. Statistically significant increases in dry mass were observed for each sample group when the irradiation dose was raised from 0 kGy to 40 kGy. The increase follows a power law behaviour as already observed in previous studies [13]. Statistically significant increases in the dry mass percentages were detected for increasing ALP concentration from 0 to 1.25 mg/mL, and again from 1.25 to 2.5 mg/mL, at each dose of electron irradiation. On the other hand, incubation in CaGP did not contribute to large differences in dry mass compared to the samples incubated in ddH_2_O.

**Figure 5:**
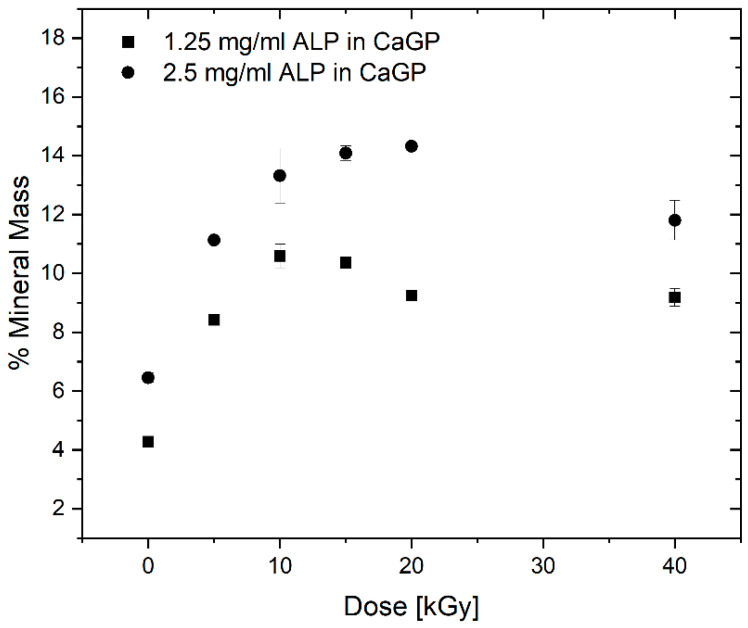
Mass percentage of mineral formed in gelatin hydrogels with ALP concentrations of 1.25 and 2.5 mg/mL (with respect to the 0 mg/mL ALP in CaGP control), at electron irradiation doses ranging from 0 to 40 kGy, mineralised for 6 days in CaGP. Results represent mean values. Error bars show standard deviation.

To determine the mass percentage of mineral formed, the difference between the dry mass percentage of mineralised and unmineralized (0 mg/mL ALP in CaGP) were calculated (see Figure 5). With increasing electron beam dose and ALP concentration, the mass percentage of formed mineral increases until a maximum at 10 and 20 kGy is reached for 1.25 and 2.5 mg/mL ALP samples, respectively, after which the mass percentage drops again.

### 3.5. Uniaxial Compression Modulus

**Figure 6:**
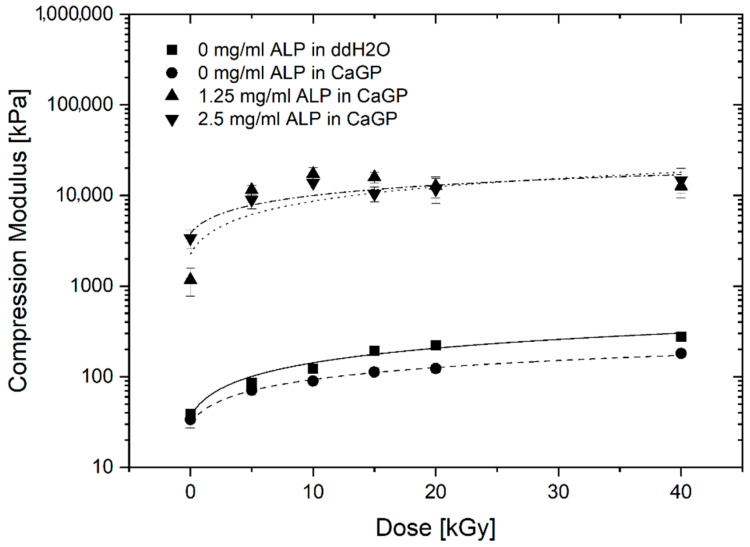
Uniaxial compression modulus of gelatin hydrogels with ALP concentrations of 0, 1.25 and 2.5 mg/mL, at electron irradiation doses ranging from 0 to 40 kGy, incubated in ddH_2_O and CaGP for 6 days. Datapoints are fitted with power law fit. Results represent mean values. Error bars show standard deviation.

The uniaxial compression moduli of gelatin hydrogels with ALP concentrations of 0, 1.25 and 2.5 mg/mL, exposed to radiation doses of 0, 5, 10, 15, 20 and 40 kGy, incubated in CaGP or ddH_2_O, are illustrated in Figure 6. For all sample groups, a statistically significant increase was identified when the samples were exposed to 40 kGy compared to the unirradiated (0 kGy) control. The increase follows a power law behaviour.

In general, 0 mg/mL ALP samples in ddH_2_O display slightly higher uniaxial compression moduli than the 0 mg/mL samples in CaGP. Raising the ALP concentration from 0 to 1.25 mg/mL resulted in a statistically significant increase in the uniaxial compression modulus at all the electron irradiation doses examined. Although the uniaxial compression moduli of samples at 2.5 mg/mL ALP were higher compared to the 1.25 mg/mL ALP samples at doses of 0 and 40 kGy of electron irradiation, this difference was not statistically significant.

### 3.6. Cytocompatibility

#### 3.6.1. Cell Culture

**Figure 7:**
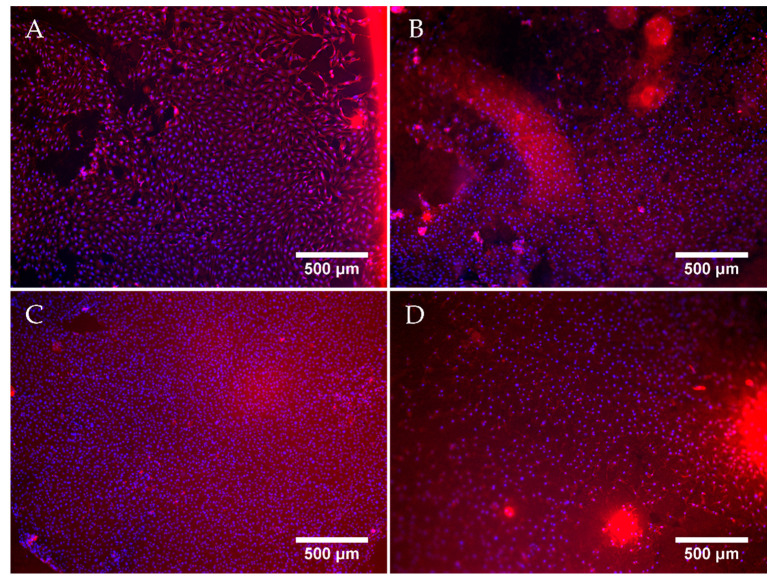
Representative fluorescent microscopy images of MG-63 osteoblast like cells cultured for 7 days on electron beam-treated (40 kGy) gelatin hydrogels with ALP concentrations of 0 mg/mL incubated in ddH_2_O (**A**), or with 0 (**B**), 1.25 (**C**) and 2.5 (**D**) mg/mL of ALP, subjected to enzymatic mineralisation in CaGP for 6 days. Cells stained by Texas Red (cell cytoplasm, red) and Hoechst (nuclei, blue). Scale bars indicate 500 µm.

Figure 7 shows exemplary fluorescent microscopy images taken after 7 days of cell culture. Successfully cultured MG-63 cells were observed on all sample groups. A confluent layer of cells covered the surface of the hydrogels of each sample group. The cells displayed a spread morphology, with clearly defined nuclei. The increase in the ALP concentration to 1.25 mg/mL did not appear to have a negative effect on cell proliferation, as the cells formed a confluent layer comparable with that on ALP-free samples. Moreover, the cell layer on samples with 1.25 mg/mL ALP was continuous and adhered well to the substrate, while the cell layer in some regions on ALP-free samples, especially those incubated in CaGP, showed a tendency to detach. However, on samples with 2.5 mg/mL ALP, the number of observed cells was considerably lower than on the other samples, as was evident from a lower concentration of the cell nuclei on the images (Figure 7), confirmed by direct cell counting (Supplementary Material, Figure S1).

#### 3.6.2. Proliferation Assay (MTS)

**Figure 8:**
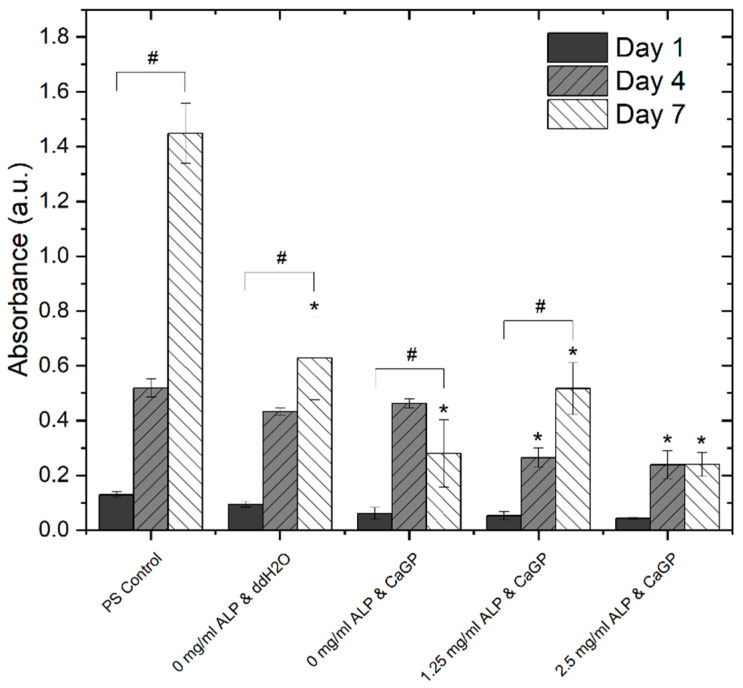
Metabolic activity of MG-63 osteoblast like cells, represented by absorbance using a MTS proliferation assay, on days 1, 4 and 7. Cells were cultured on electron beam-treated (40 kGy) gelatin hydrogels with ALP concentrations of 0, 1.25 and 2.5 mg/mL incubated in either ddH_2_O or CaGP. Polystyrene well bottoms served as a control (PS control). One-way ANOVA was performed. ‘#’ indicates statistical significance for values within sample groups (*p* < 0.05). ‘*’ indicates a statistically significant difference compared to the PS control (*p* < 0.05). Error bars show standard deviation.

In addition to direct cell counting, an MTS assay (Figure 8) was used to evaluate the effects of electron beam-treated and mineralised gelatin hydrogel samples on cellular proliferation and cell viability, and also to compare this cell behaviour with that on the standard tissue culture polystyrene (PS). No difference in metabolic activity of MG-63 cells was observed among the sample groups or in comparison with the PS control on day 1. An increase in metabolic activity was observed for each sample group on day 4. Hereby, the metabolic activity observed for 0 mg/mL ALP in ddH_2_O and 0 mg/mL ALP in CaGP samples showed no differences compared to the PS control. However, the metabolic activity for the 1.25 and 2.5 mg/mL ALP in CaGP samples demonstrated a lower value compared to the PS control. On day 7, all sample groups had lower metabolic activity compared to the PS control, suggesting reduced proliferation and increased cytotoxicity. Nevertheless, the metabolic activity of cells on CaGP samples with 1.25 mg/mL ALP tended to further increase in comparison with the value found on day 4, whereas on the other CaGP samples, this activity remained similar (samples with 2.5 mg/mL ALP) or even showed a decreasing trend (samples without ALP). From this point of view, ALP had some positive effect on final colonization of the material with cells, at least when used in a lower concentration.

These findings were, at least partly, reflected by direct counting cells on microphotographs (Figure S1 in Supplementary Material). On all tested samples, the cell number increased from day 1 to day 7, but on day 7, the cells on ALP-free CaGP-incubated samples reached a lower cell population density than on ALP-free ddH_2_O-incubated samples. On samples with 1.25 mg/mL ALP, the final cell population density became, on average, similar to that on ALP-free ddH_2_O-incubated samples, but it greatly varied between different regions on the sample, as indicated by a high standard deviation (SD). The lowest final cell population density on day 7 after seeding was found on samples with 2.5 mg/mL ALP.

## 4. Discussion

### 4.1. Formation of CaP

The successful enzymatic mineralisation of electron beam-treated gelatin hydrogels via initial incorporation of ALP and subsequent incubation in CaGP was confirmed by numerous techniques. This demonstrated that ALP retained its activity after electron beam treatment.

#### 4.1.1. Scanning Electron Microscopy (SEM)

SEM was used to provide an optical representation of ALP-induced enzymatic mineralisation of electron beam-treated gelatin hydrogels following 6 days of mineralisation in CaGP. Smooth surfaces observed for the ALP/CaGP-free control and 0 mg/mL ALP CaGP samples indicate the absence of mineralisation in this group. The sporadic deposits and inhomogeneous sections of the 0 mg/mL ALP CaGP group which were observed rather provide no evidence of mineralisation; rather, they indicate that residual CaGP remains in the hydrogel. The surface morphologies of both sample groups containing ALP (1.25 and 2.5 mg/mL) provide optical confirmation of ALP-induced mineralisation. In accordance with the findings of Douglas et al. [1], mineralisation appears to manifest as homogenously distributed globular deposits. The spatial dimensions of these deposits correspond to those observed in ALP-mineralised catechol-polyethylene glycol (cPEG), collagen and peptide amphiphile gels [1,14].

#### 4.1.2. Raman Spectroscopy

Raman spectroscopy provided direct validation of successful ALP-induced mineralisation. The absence of bands representing bone mineral in the ALP/CaGP-free sample group confirms the absence of the enzymatic mineralisation process. Similarly, the low intensity bands representing Amide III and CH_2_ deformation of gelatin hydrogels were expected. However, the low intensity peak, representative of phosphate, observed in the spectra of the 0 mg/mL ALP CaGP group was unexpected. As these samples were not exposed to the ALP-induced mineralisation procedure, this finding can most likely be explained by residual CaGP within the hydrogels, as observed in the SEM images.

The Raman spectra for both mineralised groups (1.25 and 2.5 mg/mL ALP in CaGP) show characteristics of successful mineralisation. Phosphate bands representing carbonated apatite are commonly used to confirm bone mineralisation. Therefore, these bands were expected in the Raman spectra for both sample groups exposed to ALP-induced mineralisation as observed at 560, 609 and 1000 cm^−1^. Interestingly, bands characteristic of the v1 stretching of PO_4_^3−^ were observed at ~957 cm^−1^. However, the precise position of the band characteristic of carbonated apatite’s (959 cm^−1^) is susceptible to change due to the carbonate (CO_3_^2−^) and hydrogen phosphate (HPO_4_^2−^) content of samples [15]. A high HPO_4_^2−^ content, as possessed by newly deposited or immature bone mineral, is known to shift carbonated apatite’s band to a smaller wavenumber, as observed in the present study [7,16]. Thus, the presence of this band in both ALP-containing sample groups provides confirms successful CaP mineralisation of electron beam-treated gelatin hydrogels. As the intensity of this band approximately doubled when the ALP concentration was increased from 1.25 to 2.5 mg/mL, ALP-induced mineralisation is shown to be ALP concentration-dependent. In future work, it would be interesting to compare the ratios of the regions corresponding to the mineral phase and to the organic phase, namely gelatin, in order to gain information on the ratio of mineral to organic component and hence the degree of mineralization.

#### 4.1.3. Inductively Coupled Plasma Optical Emission Spectrometry (ICP-OES)

ICP-OES was utilised to evaluate the elemental composition of the result of the mineralisation process. In general, with ALP-induced and CaGP-mediated mineralisation, the Ca- and P-concentration as well as Ca:P ratio increased significantly, providing evidence for successful incorporation of mineral phase. It was found that the Ca:P molar ratios of all sample groups are considerably lower than that of stoichiometric hydroxyapatite with a molar ratio of 1.67. This may be explained by the Ca:P ratio of the mineralisation medium (CaGP) possessing a ratio of 1.0, whereas use of media with greater molar ratios resulted in successful hydroxyapatite formation [1,14]. Additionally, variations in concentration of ALP and CaGP, as well as the hydrogel material, may lead to different mineral compositions [1]. Formation of Ca-deficient hydroxyapatite-containing HPO_4_^2−^ has been observed in previous studies where other hydrogels were mineralized with ALP [17].

#### 4.1.4. Dry Mass

The investigations on dry mass of unmineralized and ALP-mineralised gelatin hydrogels clearly show two separate effects: a dry mass dependence on electron dose as well as on mineralisation, i.e., ALP concentration. The statistically significant increase in dry mass ratio of unmineralized samples for increasing irradiation dose correlate well with gelatin crosslinking, as previously described by [13]. With increasing electron beam treatment, an increase in crosslinking is observed which leads to a reduction in water content leading to an increase in dry mass percentage.

More importantly, the ALP-induced mineralisation of the 1.25 and 2.5 mg/mL ALP hydrogels resulted in a strongly increased dry mass percentage, demonstrating the successful formation and incorporation of the mineral phase in the hydrogels. By calculating the difference between dry mass percentage of mineralized (1.25 and 2.5 mg/mL ALP in CaGP) and unmineralized (0 mg/mL ALP in CaGP) hydrogels, the mass percentage of the formed mineral can be determined. Thereby, it can be observed that the mass percentage of formed mineral is higher for the 2.5 mg/mL ALP samples confirming the ALP concentration dependence of enzymatic mineralisation. However, doubled ALP concentration does not induce the formation of twice as much mineral, which indicates that mineral formation does also depend on other factors. This is also supported by the decrease in mass percentage of formed mineral for higher electron doses (at 10 and 20 kGy for 1.25 and 2.5 mg/mL ALP samples, respectively). The decrease might be explained by the dependence of mineral formation of network structure such as pore size. With increasing electron irradiation dose, the pore size decreases [13] and the hydrogel becomes stiffer leading to a denser and less flexible network. This might hinder the incorporation of additional mineral within the hydrogel as observed in the decrease in mass percentage of formed mineral.

It would be interesting to study gelatin hydrogels without electron-beam irradiation with the aim of studying the effect of electron-beam irradiation on ALP activity in hydrogels. However, the applicability of such uncrosslinked hydrogels would be limited, as they cannot withstand the temperature required for in vitro and in vivo testing, i.e., 37 °C. In this study, we wished to verify that ALP retains activity after irradiation. Similarly, it would be interesting to investigate other radiation doses such as 30 kGy and 50 kGy in a follow-up study; this would presumably facilitate a more accurate determination of dry mass percentage as a function of dose (Figure 4).

For practical reasons, in this study it was decided to restrict ICP-OES and Raman investigations to samples irradiated with 40 kGy. Based on the results of the dry mass measurements, at lower irradiation doses, one would expect a lower level of mineral formation and hence lower signals for Ca and P from ICP-OES analysis and lower intensity bands at 957 cm^−1^ and 1000 cm^−1^, which are typical of PO_4_^3−^ groups, from Raman analysis.

Previous studies with other hydrogels have revealed that mineral formation occurs primarily in the surface regions of the hydrogel [1]. Future work could involve measurement of the thickness of the mineral layers, for example by histological techniques or micro-computer tomography.

In future work, one might consider measuring roughness, which would be increased by mineralization. Profilometry or AFM might be used, although the application of these techniques on hydrogels in the wet state is not trivial from a technical point of view.

### 4.2. Uniaxial Compression Modulus

The effect of ALP-induced mineralisation on the uniaxial compression modulus of electron beam-treated gelatin hydrogels was investigated. Increases in the irradiation dose and ALP concentration was hypothesised to lead to an increased modulus due to crosslinking and incorporation of mineralised material, respectively. For all sample groups, the uniaxial compression modulus generally increased with larger irradiation dose. This electron dose dependence was already observed for gelatin hydrogels [13] and is correlated to electron-induced crosslinking.

Hydrogels containing ALP (1.25 and 2.5 mg/mL) showed strongly increased uniaxial compression moduli, across all electron irradiation doses, compared to the 0 mg/mL ALP hydrogel groups. Although mineralisation resulted in improved mechanical properties, this does not seem to be proportional to the mass percentage of formed mineral representing the amount of incorporated mineral. More specifically, a statistically significant increase in mineral mass percentage was displayed when ALP concentration was raised from 1.25 mg/mL to 2.5 mg/mL. However, this was not mirrored in the uniaxial compression moduli. This may indicate weak interactions between the mineral phase and the polymeric phase of the electron beam-treated gelatin hydrogels after ALP-induced mineralisation [1]. The compressive modulus of cortical bone ranges from approximately 90 to 200 MPa; in this study, the compressive modulus of ALP-free samples was approximately 0.1 MPa and that of samples containing ALP was approximately 10MPa.

### 4.3. Cytocompatibility

The ability of the mineralised electron beam-treated gelatin hydrogels to promote cellular adhesion and proliferation has to be considered to evaluate if this material has potential for regenerative BTE applications. As already demonstrated, unmineralized electron beam-treated gelatin is known to be highly cytocompatible and has been proven to support cellular growth [18]. From a practical point of view, the applicability of uncrosslinked gels is limited, as they cannot withstand the temperature required for in vitro and in vivo testing, i.e., 37 °C.

The present study evaluates the interaction of electron beam-treated gelatin hydrogels containing ALP and mineralised for 6 days in CaGP with human osteosarcoma MG-63 cells. Although successful cell culture of MG-63 cells was observed on all the gelatin sample groups after 7 days, the final cell population density on day 7 after seeding was highest on the gelatin control samples (0 mg/mL ALP in ddH2O without CaGP). The cell population density was lower on samples incubated in CaGP. In accordance with this, the cell coverage of CaGP-incubated ALP-free samples on day 7 was less homogeneous and cells were prone to detachment. However, the addition of ALP at a concentration of 1.25 mg/mL restored the average cell population density to similar values as on the control samples, although with an increase in ALP concentration in samples to 2.5 mg/mL, the cell population density became lower than on the control gelatin samples. Moreover, the cell population density on ALP-containing samples varied greatly between various regions on the same sample. From this point of view, direct counting of cells on microphotographs is less reliable than indirect estimation of cell number by a test of cell metabolic activity, because this test involves all cells on the sample, while microphotographs represent only a part of the sample surface. However, it should be considered that the cell metabolic activity can be also changed by cellular processes other than cell proliferation, e.g., by cell differentiation, thus the most accurate way is to use both methods, i.e., direct cell counting and a metabolic test, together as complementary approaches. These observations are supported by the MTS assays. The metabolic activity of the cells cultured on samples incubated in CaGP was generally lower than on control samples. However, the cells on samples containing 1.25 mg/mL of ALP showed a significant increase in metabolic activity from day 1 to day 7. Moreover, the cells on samples with 1.25 mg/mL of ALP retained this activity on the same level as on day 4. On the contrary, a significant reduction in the cell metabolic activity from day 4 to 7 was observed on ALP-free CaGP samples. From this point of view, the presence of a moderate concentration of ALP in CaGP-incubated hydrogels could be considered as beneficial for colonization of these materials with bone-derived cells, although the overall cytocompatibility of the mineralised hydrogels prepared in this study was rather reduced.

However, it is unlikely that the reduction in cytocompatibility, represented by cell population density and metabolic activity, observed in the hydrogels subjected to ALP-induced mineralisation, is a result of ALP-mediated cytotoxicity as this method was already shown to be highly cytocompatible [19]. In the mentioned study, ALP was immobilized to microporous nanofibrous fibrin scaffolds in the same concentrations as used in our present study, and these scaffolds supported the proliferation of primary mouse calvarial osteoblasts to the same extent as control ALP-free fibrin scaffolds. Moreover, the ALP-enriched scaffolds increased the gene expression of markers of osteogenic differentiation in these cells, such as ALP, bone sialoprotein, type I collagen, core-binding factor I, and also osteocalcin in later culture intervals [19].

It can be also hypothesised that the adverse effects of mineralized hydrogels on cell growth could be attributed to the differences in mechanical properties of the cell culture substrate. As was shown before, the compression modulus, which can be related to the elastic modulus representing material stiffness, increases with ALP-induced mineralisation. As cells react sensitively to mechanical properties of their environment [20,21], reduction in cytocompatibility might be a result of increased material stiffness due to the incorporation of mineral material. It is known that on stiff substrates, the cells tend to spread extensively [20] and to form large and stable focal adhesion plaques [21]. Although the cell spreading and formation of focal adhesion plaques are prerequisites for the further cell survival and proliferation, these processes can also hamper the partial detachment of the cells from their adhesion substrate and the reorganization of the cytoskeleton required for mitosis (for a review, see [22]). As a result, the cells on stiffer substrates can cease their proliferation and enter their differentiation program. Increased osteogenic differentiation has been repeatedly reported in cells on stiffer substrates, including those with a mineral component [23] (for a review, see [24]), and remains to be also investigated in cells on our spontaneously mineralizing materials.

Previous studies on enzymatically mineralized hydrogels have revealed that mineral formation occurs primarily in the surface regions of the hydrogel [1]. Therefore, we are confident that CaP is present on the hydrogel surface. It should be also considered that materials containing CaP, particularly those which are calcium-deficient, can deplete calcium from the culture medium, and this phenomenon can significantly hamper the cell proliferation, especially in a conventional static cell culture system with a limited amount of cell culture medium [25], which was also employed in our present study. The cell proliferation can be improved by increasing the amount of the cell culture medium, and particularly by cultivating cells on the material in dynamic systems with medium circulation, or after their implantation in vivo [25]. Additionally, the addition of strontium to a CaP-based material significantly reduced the depletion of calcium from the culture medium and improved the proliferation of osteogenic cells [26].

Taken together, the presented results do not show optimal but promising cytocompatibility of ALP-mineralised and electron beam treated gelatin hydrogels indicating a high potential of these materials for BTE. The concentration 1.25 mg/mL ALP appears to be the most promising evaluated in this study; however, future studies might focus on lower concentrations to determine an optimum ALP concentration.

However, for optimized cytocompatibility, both the influence of mechanical stiffness on cellular behaviour as well as a potential calcium-depleting activity of the materials must be kept in mind. Future work may involve live-dead staining and a Trypan Blue exclusion assay to gain complementary data on cytocompatibility.

## 5. Conclusions

This study aimed to demonstrate and investigate the successful utilisation of electron beam irradiation to crosslink gelatin hydrogels with incorporated ALP for subsequent enzymatic mineralisation within CaGP to develop a cytocompatible and versatile material for BTE.

The present study was able to effectively demonstrate that electron beam treatment of gelatin hydrogels with incorporated ALP does not lead to inactivation of the mineralization enzyme ALP. On the contrary, ALP activity was maintained after irradiation with doses up to 40 kGy, enabling subsequent mineralisation by incubation in medium containing CaGP. As a result, CaP was successfully formed, as was demonstrated by SEM, RAMAN and ICP-OES measurements as well as dry mass determinations. Uniaxial compression tests further indicated successful mineralisation and incorporation of a mineral phase as the compression modulus increases with increasing ALP concentration. With variable irradiation dose, the uniaxial compression modulus can be further precisely tailored. Finally, the cytocompatibility of the electron beam-treated mineralised gelatin hydrogels was determined via proliferation assays showing good cytocompatibility, which has to be improved in order to achieve excellent cytocompatibility. However, the presented study indicates the high potential of electron beam treatment of ALP-gelatin hydrogels for bone tissue engineering as uncrosslinked gelatin hydrogels themselves are not applicable in vivo or in vitro; however, after electron beam treatment and ALP-mediated mineralisation, they can be applied as a thermally stabilised biomaterial for bone mimicry and potential replacement.

## Data Availability

The data presented in this study are available on request from the corresponding author.

## Supplementary Materials

The following are available online at https://www.mdpi.com/article/10.3390/jfb12040057/s1, Figure S1: The population density of MG-63 osteoblast like cells on days 1, 4 and 7 after seeding, obtained by direct counting cells on microphotographs. Cells were cultured on electron beam-treated (40 kGy) gelatin hydrogels with ALP concentrations of 0, 1.25 and 2.5 mg/ml, incubated in either ddH_2_O or CaGP. Mean ± Standard Deviation (A) or mean only (B) from 2–4 representative microphotographs (each representing an area of approx. 40 mm^2^) for each experimental group and time interval.
