# Supplementary material for: Electron Beam-Treated Enzymatically Mineralized Gelatin Hydrogels for Bone Tissue Engineering
Source: J Funct Biomater. 2021 Oct 8;12(4):57. doi: 10.3390/jfb12040057 (PMC8544455; doi:10.3390/jfb12040057)
Supplement: Supplementary file 1 [file jfb-12-00057-s001.zip › jfb-1327500-supplementary.pdf]

Electron Beam-Treated Enzymatically Mineralized Gelatin Hydrogels for Bone Tissue Engineering

Stefanie Riedel [1,2,*], Daniel Ward [3], Radmila Kudláčková [4], Karolina Mazur [5], Lucie Bačáková [4], Jemma G. Kerns [6], Sarah L. Allinson [3], Lorna Ashton [7], Robert Koniezcny [1], Stefan G. Mayr [1,2] and Timothy E. L. Douglas [8,9,*]

[1] Leibniz Institute of Surface Engineering (IOM), Permoserstraße 15, 04318 Leipzig, Germany; robert.konieczny@iom-leipzig.de (R.K.); stefan.mayr@iom-leipzig.de (S.G.M.)
[2] Division of Surface Physics, Faculty of Physics and Earth Science, Leipzig University, Linnéstraße 5, 04103 Leipzig, Germany
[3] Division of Biomedical and Life Sciences (BLS), Faculty of Health and Medicine, Furness College, Lancaster University, Lancaster LA1 4YG, UK; dannywlmr9@hotmail.co.uk (D.W.); s.allinson@lancaster.ac.uk (S.L.A.)
[4] Department of Biomaterials and Tissue Engineering, Institute of Physiology of the Czech Academy of Sciences, Vídeňská 1083, 142 00 Prague, Czech Republic; KudlackovaRadmila@seznam.cz (R.K.); Lucie.Bacakova@fgu.cas.cz (L.B.)
[5] Faculty of Materials Engineering and Physics, Institute of Materials Engineering, Tadeusz Kosciuszko Cracow University of Technology, al. Jana Pawła II 37, 31-864 Cracow, Poland; mazur117@o2.pl
[6] Lancaster Medical School, Faculty of Health and Medicine, Lancaster University, Lancaster LA1 4YW, UK; j.kerns@lancaster.ac.uk
[7] Chemistry Department, Lancaster University, Lancaster LA1 4YB, UK; l.ashton@lancaster.ac.uk
[8] Engineering Department, Lancaster University, Lancaster LA1 4YW, UK
[9] Materials Science Institute (MSI), Lancaster University, Lancaster LA1 4YW, UK
* Correspondence: stefanie.riedel@iom-leipzig.de (S.R.); t.douglas@lancaster.ac.uk (T.E.L.D.).

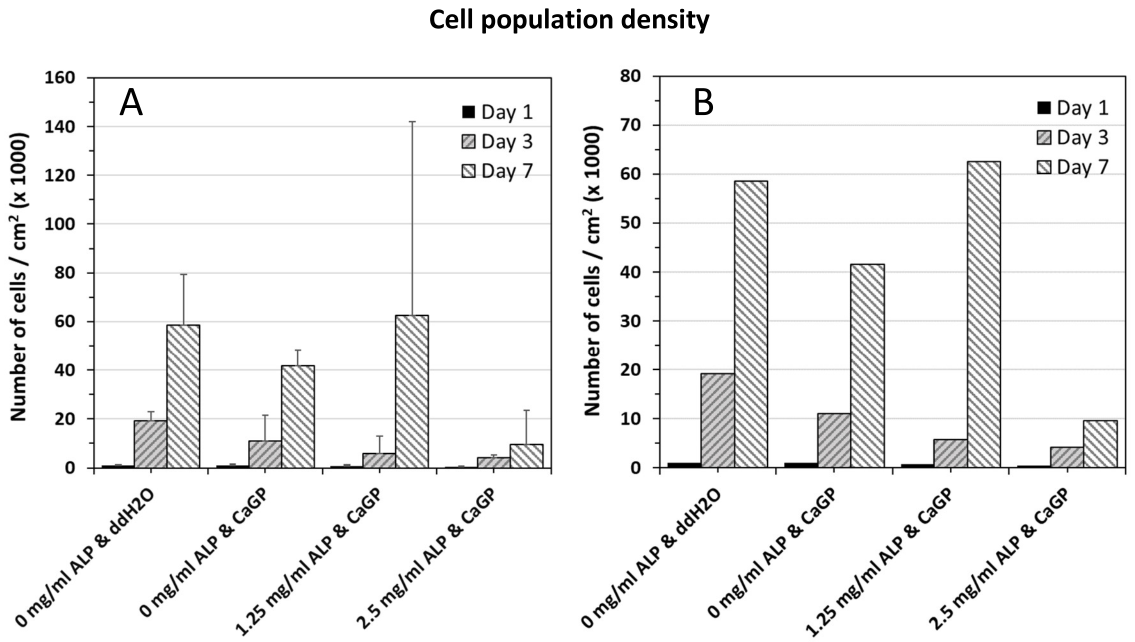

Figure S1. The population density of MG-63 osteoblast like cells on days 1, 4 and 7 after seeding, obtained by direct counting cells on microphotographs. Cells were cultured on electron beam-treated (40 kGy) gelatin hydrogels with ALP concentrations of 0, 1.25 and 2.5 mg/ml, incubated in either ddH$_2$O or CaGP. Mean ± Standard Deviation (**A**) or mean only (**B**) from 2-4 representative microphotographs (each representing an area of approx. 40 mm^2) for each experimental group and time interval.
